# Trigger point injections and dry needling can be effective in treating long COVID syndrome-related myalgia: a case report

**DOI:** 10.1186/s13256-021-03239-w

**Published:** 2022-01-17

**Authors:** Mengyi Zha, Kristina Chaffee, Jude Alsarraj

**Affiliations:** 1 Columbia Basin Health Association, 1515 E Columbia St, Othello, WA 99344 USA; 2 Targeted Rural Underserved Track, School of Medicine, University of Washington, Seattle, WA USA; 3 University of Washington School of Medicine, Seattle, WA USA

**Keywords:** Case report, Long COVID syndrome, Myofascial pain, Trigger point injections, Dry needling

## Abstract

**Introduction:**

Myofascial pain is a complex health condition that affects the majority of the general population. Myalgia has been recognized as a symptom of long COVID syndrome. The treatment for long COVID syndrome-related myalgia lacks research. Dry needling is a technique that involves the insertion of a needle into the tissue of, or overlaying, a pain point. Wet needling is the addition of an injection of an analgesic substance such as lidocaine while performing needling. Both dry and wet needling have are practiced as treatment modalities for myofascial pain. Limited literature exists to define long COVID syndrome-related myalgia and its relation to myofascial pain, or to examine the utility of needling techniques for this pain. We report a case of dry and wet needling as effective treatments for long COVID-related myofascial pain.

**Case presentation:**

A 59-year-old, previously healthy Hispanic male with no comorbid conditions was diagnosed with COVID-19 pneumonia. The patient suffered moderate disease without hypoxia and was never hospitalized. Three months later, the patient continued to suffer from symptoms such as exertional dyspnea, “brain fog,” and myalgia. An extensive multisystem workup revealed normal cardiac, pulmonary, and end organ functions. The patient was then diagnosed with long COVID syndrome. The nature and chronicity of the patient’s myalgia meet the criteria for myofascial pain. Both wet and dry needling were used to treat the patient’s myofascial pain, with good short- and long-term therapeutic effects.

**Conclusions:**

COVID-19 infection has been shown to exacerbate preexisting myofascial pain syndrome. Our case report indicates that long COVID syndrome-related myalgia is likely a form of new-onset myofascial pain. Additionally, both wet and dry needling can be utilized as an effective treatment modality for this pain syndrome, with short- and long-term benefits.

## Introduction

Myofascial pain (MP) is a complex health condition that affects many patients, and is thought to occur from muscle overuse, trauma, and psychological stress [1]. The loci of the pain are termed “trigger points,” which are tender spots in a taut muscle band. When pressure is applied to these trigger points, it produces a pain response [2]. Although trigger points can be reliably detected on ultrasound as hypoechoic foci within the muscle [3, 4], the exact mechanism by which trigger points form remains debated [1, 5]. While not interchangeable, fibromyalgia (FM) and MP syndrome have overlapping diagnostic criteria [6].

Post-acute COVID or long COVID syndrome (LCS) has become increasingly recognized as a prevalent disease and long-term sequela of COVID-19 survivors [7–9]. Common symptoms include fatigue, dyspnea, palpitations on minimal exertion, cognitive impairment or “brain fog,” sleep disturbances, digestive issues, mood disorders, headache, and myalgia [10, 11]. Myalgia has been commonly associated with LCS, either in the sense of new pain or exacerbation of preexisting pain [12–15]. Interestingly, myalgia following SARS-CoV-2 infection seems to be independent of the severity of the initial infection [8]. In general, a multidisciplinary approach is recommended for the management of LCS [9, 10, 16]. However, effective management of this lingering myalgia remains to be explored.

Dry needling (DN) is an umbrella term to describe an array of different techniques that generally involve the insertion of a needle into the tissue of, or overlaying, a trigger point [17]. It is a relatively new, but increasingly widely used technique to treat myofascial pain [18–20]. In some studies, DN is shown to reduce stiffness and tone, improve contractile properties of certain muscles, and decrease pressure pain perception [21, 22]. The pain reduction effect of DN is apparent both immediately after treatment and at an interval of 4 weeks in multiple body regions [23–25]. Wet needling (WN), or trigger point injection, is the additional injection of an analgesic substance, such as lidocaine, while performing needling. Long- and short-term efficacy varies between the two techniques [26]. However, acute pain relief tends to be greater with WN [27–29].

Limited literature exists to define LCS-related myalgia and its relation to MP, though treatment options are urgently needed. Although DN and WN are both effective treatment modalities for MP, their utility for LCS-related myalgia have not been examined.

## Case presentation

We report a case of WN and DN as effective treatments for LCS-related MP.

In late June 2020 (T_0_), a 59-year-old previously robust and healthy Hispanic male with no prior chronic illness, presented with symptoms consistent with COVID-19 illness, and was diagnosed with by an RT-PCR test for SARS-CoV-2. His vital signs were within normal limits, with an SpO_2_ of 95%. Chest X-ray showed multifocal pneumonia. He then returned home for supportive management of his acute illness.

At T_0_+3 weeks, the patient continued to have difficulty breathing, chest pain, weakness, headaches, and diffuse myalgia. The patient’s vital signs were normal, with an SpO_2_ of 96%. Repeat chest X-ray showed unchanged multifocal pneumonia. D-dimer was 4960 ng/mL, white blood cell (WBC) count was within normal limits, with normal absolute lymphocyte count. The patient did not meet inpatient admission criteria and was therefore encouraged to rest and recover at home. At T_0_+6 weeks, during a phone visit, the patient described his persistent profound myalgia and associated weakness. He gave an example that he was not even able to open a water bottle without pain.

At T_0_+3 months, the patient presented to the clinic for disability paperwork owing to his inability to complete daily living activities caused by his persistent exertional dyspnea, “brain fog,” and myalgia. Today, we know that LCS has a significant impact on a patient’s employment [16]. Repeat chest X-ray showed normal findings. Pulmonary function test was normal. Electrocardiogram (EKG), troponin, B-type natriuretic peptide, hemoglobin A1C, and thyroid stimulating hormones were within normal limits. Echocardiogram showed normal left ventricular function and no significant valvular disease. Nuclear stress test was negative for arrhythmia or coronary artery ischemia. Additionally, depression screening was negative.

The patient’s symptoms persisted. At T_0_+6 months, the diagnosis of LCS was made, according to the few studies available at the time [11]. The chronicity of the patient’s diffuse myalgia qualified his pain as chronic pain, and the locations were typical for MP syndrome and FM: neck, shoulder, upper back, bilateral posterior upper arms, and posterior lower legs. His widespread pain index was greater than 7. An 11-point numeric Likert pain score was used to document pain severity, with 0 being “no pain at all” and 10 being “the worst pain I have had.” The patient’s pain score was 6. At this point, the patient met the American College of Rheumatology preliminary diagnostic criteria for FM [30]. He scored 88 out of 100 on the Fibromyalgia Impact Questionnaire (FIQ), representing severe impact of his pain syndrome on his physical functioning, mood, and overall well-being.

At the visit at T_0_+6 months, WN with 1% lidocaine without epinephrine was performed on six points, using a 25 gauge, 1.5 inch needle: four in the neck and shoulder regions, and one on each side of posterior triceps. In all of our WN practice, each trigger point receives 1 ml lidocaine. Immediately after the injections, the patient stated an improvement in pain, with a pain score of 0–1. The benefit of this WN session lasted for 2 weeks, with improved daily living activity function and pain intensity. At this time, the patient was prescribed 30 mg duloxetine daily. On 2 week follow-up, the patient rated his pain score to be six again. The patient then received five more WN injections: three in the neck and shoulder regions, and one in each posterior tricep. Immediately after injection, the patient commented he only had “2% of pain left,” indicating a pain score of 0–1. At 2 weeks follow-up after the second WN session, the patient only reported pain in the bilateral posterior triceps region, with a pain score of two. A third WN session was performed with two injections: one in each posterior tricep. Immediately after the injections, the patient had no more pain. One month later, at T_0_+7 months, the patient received four more WN injections: one in the left posterior distal thigh, one in the right upper posterior deltoid, and two in the neck and shoulder regions. His pain score reduced to 0 immediately after the injection, from 2 on presentation. The patient reported feeling ready to return to work.

At T_0_+12 months, the patient returned to clinic with worsening LCS: numbness and tingling of hands and feet for 4 weeks, leg cramping, forgetfulness, and diffuse myalgia. Since his last visit, the patient had experienced significantly increased psychosocial stress, including COVID-19-related deaths in the family. He felt that the stress exacerbated his symptoms.

At this point, DN was trialed with a 21 gauge, 1 inch needle. A total of 10 points were needled: four in the neck and upper back region, one in each posterior tricep, and two in each posterior calf muscle. Immediately, after the first DN session, the patient had a reduction in pain score, from 8 to 2. Two weeks later, the patient reported pain score of 2, and a second session of DN was done. The same 10 points were needled as in the previous visit. The patient subsequently reported no more pain after DN.

At the time of this case report, during the last documented follow-up appointment (T_0_+18 months, September 2021), the patient remained pain free, with a pain scale of 0. No adverse reaction to WN or DN treatments were reported.

## Discussion and conclusions

In the case we describe above, the patient had no preexisting conditions that could explain the new onset of myalgia. The most feasible explanation of his lingering myalgia was LCS. The nature of the pain and the loci of trigger points of the patient’s pain indicate that the mechanism was probably MP. Additionally, psychological stress exacerbated the patient’s pain, which has been seen in MP syndrome [31].

Although no direct connection has been made to support new-onset MP syndrome caused by LCS, a recent case series demonstrated a correlation between worsening MP syndrome trigger point pain after COVID-19 infection. The authors indicated that COVID-19 infection could elicit physical and psychological stress, which could lead to more trigger points in patients with MP syndrome [32]. Our case report adds to the possibility of the development of new-onset MP as part of LCS.

If this patient did not have LCS as an explanation for his pain, his pain syndrome would have met the diagnostic criteria for FM. It is also safe to assume that, in clinical practice, many LCS will be misdiagnosed as FM. There are most likely overlapping pathogenesis between the two pain syndromes. Therefore, the FIQ might provide a good method to measure the impact of LCS-related MP.

This report has a few limitations. First, the rheumatologic causes of this patient’s lingering symptoms was not investigated so, although unlikely, such causes cannot be completely ruled out for his pain. Second, the patient was started on duloxetine for pain management prior to starting DN or WN. Although duloxetine does not explain the immediate improvement of the patient’s pain scores after needling treatments, it is a confounding factor for the patient’s longer-term pain improvement. Last, placebo effect of needling treatments cannot be ruled out from a single case report. Larger studies, ideally in the form of randomized control trials, are needed to validate the effectiveness of DN and WN for LCS-related myalgia.

With over 220 million people having recovered from COVID-19 worldwide, including 37 million in the USA alone, LCS will likely become a new form of common chronic illness [9]. Most symptoms will be managed in primary care settings [7]. Effective treatment modality for this disease needs urgent investigation. However, there is very little information on the treatment of LCS-related myalgia in existing guidelines [10, 15, 16]. From our experience, we conclude that WN and DN can be effective and safe ways to treat LCS-related myalgia in the primary care setting, with both short- and long-term benefits. This case report is, so far, the only report that provides insight on the therapeutic effects of needling techniques that can be easily employed in the primary care setting.

## Data Availability

The deidentified data that support the findings of this report are available on request from the corresponding author, MZ. The data are not publicly available due to HIPPA.

## Abbreviations

MP: Myofascial pain
FM: Fibromyalgia
LCS: Long COVID syndrome
T_0_: Initial time of symptom onset
WN: Wet needling
DN: Dry needling

## References

[1] DG Simons 1996 Clinical and etiological update of myofascial pain from trigger points J Musculoskel Pain. 4 1–2 93 122

[2] EA Tough AR White S Richards J Campbell 2007 Variability of criteria used to diagnose myofascial trigger point pain syndrome–evidence from a review of the literature Clin J Pain 23 3 278 286 10.1097/AJP.0b013e31802fda7c 17314589

[3] N Taheri F Okhovatian A Rezasoltani M Karami SM Hosseini HK Mohammadi 2016 Ultrasonography in diagnosis of myofascial pain syndrome and reliability of novel ultrasonic indexes of upper trapezius muscle Ortop Traumatol Rehabil 18 2 149 154 10.5604/15093492.1205022 28155823

[4] JJ Ballyns JP Shah J Hammond T Gebreab LH Gerber S Sikdar 2011 Objective sonographic measures for characterizing myofascial trigger points associated with cervical pain J Ultrasound Med 30 10 1331 1340 10.7863/jum.2011.30.10.1331 PMC3493620 21968483

[5] MS Jafri 2014 Mechanisms of Myofascial Pain Int Sch Res Notices. 2014 9 10.1155/2014/523924 PMC4285362 25574501

[6] S Bourgaize I Janjua K Murnaghan S Mior J Srbely G Newton 2019 Fibromyalgia and myofascial pain syndrome: Two sides of the same coin? A scoping review to determine the lexicon of the current diagnostic criteria Musculoskeletal Care 17 1 3 12 10.1002/msc.1366 30350334

[7] M Mendelson J Nel L Blumberg 2020 Long-COVID: An evolving problem with an extensive impact S Afr Med J 111 1 10 12 10.7196/SAMJ.2020.v111i11.15433 33403997

[8] JM Anaya M Rojas ML Salinas 2021 Post-COVID syndrome. A case series and comprehensive review Autoimmun Rev 89 102947 10.1016/j.autrev.2021.102947 PMC8428988 34509649

[9] DL Sykes L Holdsworth N Jawad P Gunasekera AH Morice MG Crooks 2021 Post-COVID-19 symptom burden: what is long-COVID and how should we manage it? Lung 199 2 113 119 10.1007/s00408-021-00423-z PMC7875681 33569660

[10] H Crook S Raza J Nowell M Young P Edison 2021 Long covid-mechanisms, risk factors, and management BMJ 374 1648 10.1136/bmj.n1648 34312178

[11] A Nath 2020 Long-Haul COVID Neurology 95 13 559 560 10.1212/WNL.0000000000010640 32788251

[12] C Fernández-de-Las-Peñas J Rodríguez-Jiménez S Fuensalida-Novo 2021 Myalgia as a symptom at hospital admission by severe acute respiratory syndrome coronavirus 2 infection is associated with persistent musculoskeletal pain as long-term post-COVID sequelae: a case-control study Pain 678 8 10.1097/j.pain.0000000000002306 33863864

[13] F Bakılan İG Gökmen B Ortanca 2021 Musculoskeletal symptoms and related factors in postacute COVID-19 patients Int J Clin Pract 8 e14734 10.1111/ijcp.14734 PMC8420386 34387911

[14] S Zayet H Zahra PY Royer 2021 Post-COVID-19 syndrome: nine months after SARS-CoV-2 infection in a cohort of 354 patients: data from the first wave of COVID-19 in Nord Franche-Comté Hospital France. Microorganisms. 9 8 10.3390/microorganisms9081719 PMC8401026 34442798

[15] Chippa V, Aleem A, Anjum F. Post Acute Coronavirus (COVID-19) Syndrome. In: *StatPearls.* Treasure Island (FL): StatPearls Publishing Copyright © 2021, StatPearls Publishing LLC.; 2021.

[16] OL Aiyegbusi SE Hughes G Turner 2021 Symptoms, complications and management of long COVID: a review J R Soc Med 114 9 428 442 10.1177/01410768211032850 PMC8450986 34265229

[17] J Dommerholt 2011 Dry needling - peripheral and central considerations J Man Manip Ther 19 4 223 227 10.1179/106698111X13129729552065 PMC3201653 23115475

[18] L Kalichman S Vulfsons 2010 Dry needling in the management of musculoskeletal pain J Am Board Fam Med 23 5 640 646 10.3122/jabfm.2010.05.090296 20823359

[19] C Unverzagt K Berglund JJ Thomas 2015 Dry needling for myofascial trigger point pain: a clinical commentary Int J Sports Phys Ther 10 3 402 418 PMC4458928 26075156

[20] AD Furlan MW Tulder van DC Cherkin 2005 Acupuncture and dry-needling for low back pain Cochrane Database Syst Rev 1 001351 10.1002/14651858.CD001351.pub2 PMC12145953 15674876

[21] DM Kietrys KM Palombaro E Azzaretto 2013 Effectiveness of dry needling for upper-quarter myofascial pain: a systematic review and meta-analysis J Orthop Sports Phys Ther 43 9 620 634 10.2519/jospt.2013.4668 23756457

[22] A Pérez-Bellmunt O Casasayas-Cos C López-de-Celis 2021 Effects of dry needling of latent trigger points on viscoelastic and muscular contractile properties: preliminary results of a randomized within-participant clinical trial J Clin Med 10 17 10.3390/jcm10173848 PMC8432234 34501299

[23] J Sánchez-Infante A Bravo-Sánchez F Jiménez J Abián-Vicén 2021 Effects of dry needling on mechanical and contractile properties of the upper trapezius with latent myofascial trigger points: a randomized controlled trial Musculoskelet Sci Pract. 56 102456 10.1016/j.msksp.2021.102456 34507046

[24] L Liu QM Huang QG Liu 2015 Effectiveness of dry needling for myofascial trigger points associated with neck and shoulder pain: a systematic review and meta-analysis Arch Phys Med Rehabil 96 5 944 955 10.1016/j.apmr.2014.12.015 25576642

[25] R Boyles R Fowler D Ramsey E Burrows 2015 Effectiveness of trigger point dry needling for multiple body regions: a systematic review J Man Manip Ther 23 5 276 293 10.1179/2042618615Y.0000000014 PMC4768380 26955257

[26] CZ Hong 1994 Lidocaine injection versus dry needling to myofascial trigger point. The importance of the local twitch response Am J Phys Med Rehabil 73 4 256 263 10.1097/00002060-199407000-00006 8043247

[27] B Fogg S Daly 2021 Are trigger point injections with anesthetic (wet needling) more effective than trigger point injections without anesthetic (dry needling) in treating patients with myofascial pain? Evidence-Based Practice. 24 8 24 25

[28] DA Ibrahim HA Abdelrahem 2019 Cervical region trigger point Injection with dry needling versus wet needling by lidocaine in geriatric population: a comparative study Ain-Shams J Anesthesiol 11 1 16

[29] SA Raeissadat SM Rayegani F Sadeghi S Rahimi-Dehgolan 2018 Comparison of ozone and lidocaine injection efficacy vs dry needling in myofascial pain syndrome patients J Pain Res 11 1273 1279 10.2147/JPR.S164629 PMC6029589 29988746

[30] Don L, Goldenberg M. Clinical manifestations and diagnosis of fibromyalgia in adults. UpToDate. https://www.uptodate.com/contents/clinical-manifestations-and-diagnosis-of-fibromyalgia-in-adults?search=fibromyalgia%20diagnosis&sectionRank=2&usage_type=default&anchor=H198274414&source=machineLearning&selectedTitle=1~150&display_rank=1#H198274414. 2020. Accessed 10/23, 2021.

[31] A Saxena M Chansoria G Tomar A Kumar 2015 Myofascial pain syndrome: an overview J Pain Palliat Care Pharmacother 29 1 16 21 10.3109/15360288.2014.997853 25558924

[32] J Patel S Javed 2021 Myofascial pain syndrome and SARS-CoV-2: a case series Pain Manag 8 9 10.2217/pmt-2021-0044 PMC8491650 34601951

